# Treatment of Epilepsy Associated with Common Chromosomal Developmental Diseases

**DOI:** 10.1515/biol-2020-0003

**Published:** 2020-02-28

**Authors:** Magdalena Budisteanu, Claudia Jurca, Sorina Mihaela Papuc, Ina Focsa, Dan Riga, Sorin Riga, Alexandru Jurca, Aurora Arghir

**Affiliations:** 1 University of Oradea, Faculty of Medicine and Pharmacy, Preclinical Department, Oradea Romania; 2 Prof. Dr. Alexandru Obregia” Clinical Hospital of Psychiatry, Bucharest Romania; 3 ”Victor Babes“ National Institute of Pathology, Bucharest Romania; 4 ”Titu Maiorescu” University – Faculty of Medicine, Bucharest Romania; 5 ”Carol Davila” University of Pharmacy and Medicine, Bucharest Romania

**Keywords:** chromosomal diseases, epileptic seizures, antiepileptic drugs

## Abstract

Chromosomal diseases are heterogeneous conditions with complex phenotypes, which include also epileptic seizures. Each chromosomal syndrome has a range of specific characteristics regarding the type of seizures, EEG findings and specific response to antiepileptic drugs, significant in the context of the respective genetic etiology. Therefore, it is very important to know these particularities, in order to avoid an exacerbation of seizures or some side effects. In this paper we will present a review of the epileptic seizures and antiepileptic treatment in some of the most common chromosomal syndromes.

## 1 Introduction

Neurogenetic developmental diseases represent a group of genetic heterogeneous conditions characterized by intellectual disability, dysmorphic features, behavior problems (autism, hyperactivity, etc.), neurologic and/or psychiatric diseases, and different malformations. Epileptic seizures are common features of most of these conditions, with serious implications on patient management. Early diagnosis and a proper treatment of epilepsy in these patients are essential for a good outcome and a better quality of life. In this paper we review the impact of different genetic defects on therapeutic strategy of epileptic seizures, providing as examples the most common chromosomal developmental syndromes: Down syndrome, Angelman syndrome, Prader-Willi syndrome, and 22q11.2 deletion syndrome.

## 2 Down syndrome

Down syndrome (DS) is the most common genetic cause of intellectual disability with a prevalence of 1 in 700 -1000 newborns [1]. In 90% of DS cases it is caused by the presence of a supernumerary chromosome 21. Other chromosomal mechanisms leading to partial or full trisomy 21 are unbalanced translocations, Robertsonian translocations, duplications, mosaicism. The clinical presentation is characterized by psychomotor delay (usually mild to moderate), specific facial dysmorphic features and limb anomalies (affecting mainly the hands), hypotonia, and different visceral anomalies (heart defects, digestive anomalies, eyes, ears, or teeth defects, etc.).

The prevalence of epilepsy in people with DS has been reported to range from 1% to 13% [1]. Different types of epileptic seizures have been reported, including infantile spasms (IS), focal seizures (FS), generalized tonic-clonic seizures (GS), absences etc. [1]. The mechanism of the seizures is not completely understood, so far. Studies focused on delineation of Down syndrome critical region (DSCR) based on extensive genotype-phenotype correlations [2, 3], suggested the involvement of more than a single chromosome 21 critical region in generating the entire phenotype. Thus, it seems more relevant to search for dosage-sensitive genes contributing to specific clinical manifestations. Among these genes, some proposed contributors to DS-associated brain phenotypes were *KCNJ6* (potassium inwardly rectifying channel subfamily J member 6)*, RCAN1* (regulator of calcineurin 1), *DYRK1A* (dual specificity tyrosine phosphorylation regulated kinase 1A), *SIM2* (SIM bHLH transcription factor 2), *DSCAM* (DS cell adhesion molecule)*, GRIK1* (glutamate ionotropic receptor kainate type subunit 1)*, APP* (amyloid beta precursor protein)*, S100B* (S100 calcium binding protein B)*, SOD1* (superoxide dismutase 1) [4, 5, 6, 7, 8, 9, 10]. In some cases, seizures have been attributed to structural anomalies of the brain or related to medical complications, such as congenital cardiovascular anomalies (Moyamoya’s disease), intracranial bleed and chemotherapy related neurotoxicity, bacterial and viral neurological infections [11, 12].

Studies on animal models revealed several risk factors including an abnormal neuronal structure, a decreased neurotransmission inhibition, a hyperexcitable membrane in ion channels, an increased GABA-B receptor activity [1]. For the treatment of IS in children with DS, different schedules have been used. Presently, the first-line therapy of this type of seizures includes vigabatrin (VGB) and steroids or ACTH [13, 14], with a good control of seizures in almost half of children with DS and IS [15]. VGB represents an efficient drug for IS control, indicated mainly in tuberous sclerosis patients; however, a special care should be taken regarding the severe visual field defects due to retinal toxicity, reported with different frequencies in patients treated with VGB [16,17].

Regarding the treatment with steroids, there are some controversies concerning their efficacy. Some authors reported a better response in children with IS and DS compared to children without DS [18]. In cases with infantile spasm without DS the outcome is, generaly, poor, depending on the underlying cause; there is a high risk of epilepsy, severe intellectual disability and autism in these children, especialy in cases with structural brain anomalies (such in tuberous sclerosis) [19].

However, other authors found a worse outcome for steroid treatment in IS in children with DS [20]. Another important aspect of the treatment with steroids is related to the serious side effects (increased weight or obesity, Cushing syndrome, behavioral disturbance, hypertension, hypokaliemia, femoral fracture, cardiac decompensation) of this therapy, taking into account that high doses are needed for seizures control in these patients [21]. Valproate (VPA), phenobarbital (PHB), topiramate (TPM) and levetiracetam (LVT) were also used with good effects in some children with DS and IS [12].

Regarding the treatment of other types of seizures (focal seizures, generalized seizures, absences), VPA is recommended as first-line option, alone or in association with lamotrigine (LTG) [12].

Recently, Deidda et al. reported a positive effect of bumetanide (inhibitor of NKCC1 co-transporter) on epileptic seizures in DS mouse model by reversing the excitatory GABAA receptor signaling and restoring the inhibitory GABAergic currents [22]. Additionally, bumetanide enhanced the learning and memory performance by restoring the synaptic plasticity [22].

When we treat epilepsy in children with DS and other neurogenetic conditions, both the effect of seizures and of the antiepileptic drugs (AEDs) on child neurodevelopment should be taken into account. Thus, Goldberg et al. showed that children with DS and IS had a poor neurodevelopment despite the fact that seizures control was good [13]. On the other hand, Eiserman et al. reported delayed neurodevelopment and autistic behavior in children with DS and epilepsy who started the antiepileptic treatment with a delay of more than two months [23].

In DS patients over 50 years of age, late myoclonic seizures have been observed, especially in cases with dementia [24]. Moreover, epileptic seizures exacerbate the impairment of cognitive functions [25]. This type of seizures can be successfully treated with new AEDs, such as LVT and TPM; VPA was, also, used with good effect [1, 25]. However, the risk of side effects in these patients is higher, including those with impact on central nervous system: somnolence, dizziness, distractibility [26]. TPM should be used with caution because it can increase the cognitive decline [24]. Also, the AEDs which act on sodium channel and can aggravate myoclonic seizures, such as carbamazepine (CBZ) and phenytoin (PHT), should be avoided [25].

## 3 Angelman syndrome

Angelman syndrome (AS) is a severe genetic neurodevelopmental disease secondary to the loss of function of E6-AP ubiquitin ligase (*UBE3A*) gene caused by one of these four mechanisms: deletion of chromosome 15q11–q13 region of maternal origin (75%), paternal uniparental disomy (5-10%), an imprinting defect (5-10%) or point mutation in the maternal origin allele of *UBE3A* [27]. The prevalence of AS among children and young adults is approximately 1 in 12000-20000; with males and females equally affected [28]. AS is characterized by severe intellectual disability, specific facial dysmorphic features, ataxia, severe speech delay, a characteristic behavioral phenotype (happy disposition, sleep disorder, water attraction). Epilepsy is a common feature (80 to 95%) in AS, often with onset before the age of 3 years [29, 30]. The mechanism of the seizures is not very clear, the haploinsufficiency of a cluster of GABA receptors including GABRB3 in the distal end of 15q chromosome could be involved [31, 32, 33]. *UBE3A* gene product is part of the ubiquitin protein degradation system, being involved in recognition and digestion of ubiquitintargeted proteins at proteasome level. As loss of function mutations in maternal *UBE3A* allele lead to AS phenotype, it can be hypothesized that improper ubiquitin substrate regulation in those tissues where the UBE3A expression is dependent on the maternal allele contributes to the pathogenesis of the disorder [34]. Recent studies on mouse model demonstrate that *Ube3a* loss from GABAergic neurons produces AS-like EEG changes, enhances seizure susceptibility and severity [35].

Patients with AS show a characteristic pattern on electroencephalogram (EEG) with large-amplitude slow-spike waves of 1–2 or 4–6 Hz [36, 37]. Different types of seizures can be observed: FS, GS, tonic and atonic seizures, myoclonic, atypical absences [31, 38]. In many cases the seizures are resistant to AEDs, with a significant impact on life quality of these patients.

Different AEDs can be used in patients with AS. VPA and clonazepam (CLZ) are the most frequently used AEDs; LVT, LTG and clobazam (CLB) have also been commonly prescribed having less adverse effects [39, 40]. Recurrent myoclonic status epilepticus (SE) can be treated with VPA and ethosuximide (ESM) [41]. In some cases with refractory epilepsy TPM and ESM were effective [14]. CBZ, oxcarbazepine (OCZ) and VGB should be avoided in patients with AS because they can lead to worsening the seizures [29]. As seizures in AS are typically refractory to therapy, many patients need more than one AED for seizures control.

An important aspect of antiepileptic therapy in patients with AS is related to the side effects, especially those with neuropsychiatric impact (tremor, imbalance, motor regression). Thus, development of new alternative methods to treat epileptic seizures in this disease is crucial. Several studies showed that ketogenic diet (KD) and low-glycemic index treatment (LGIT) are efficient and well-tolerated in patients with AS, due to a decrease of the neuronal excitability [42, 43, 44]. Studies on AS mouse models showed that oral administration of ketone esters (KE) induces therapeutic ketosis and has anticonvulsivant effect and improves motor and cognitive functions by increasing GABA/glutamate ratio [44]. In AS children with refractory epilepsy KD proved, also, to have a good effect [41]. LGIT was used to treat epilepsy in children with AS with highly efficacy [43]. Moreover, LGIT was more acceptable to the children and easier to integrate into daily meals than KD. The side effects of LGIT therapy include constipation and metabolic acidosis, which should be taken into consideration for the management plan of patients with AS [43].

Myoclonus and non convulsive status epilepticus are other typical epileptic manifestations of AS. In children with AS non convulsive status epilepticus (NCSE) was reported in about 50% of cases and may include atypical absences, decreased alertness, hypotonia, atonic head drop, myoclonic movements, motor or developmental regression, somnolence or increased fatugability. NCSE can be triggered by different situations, such as infections, tapering of antiepileptic drugs, allergies or constipation. EEG shows slow sharp-waves discharges with high amplitude, especially on frontal derivations.

For distal myoclonus, an effective drug proved to be Piracetam [45], while non convulsive status epilepticus showed a variable response to benzodiazepines and corticosteroids [46].

## 4 Prader–Willi syndrome

Prader–Willi syndrome (PWS) is a rare genetic condition (prevalence of 1 in 10,000 – 1 in 30,000), characterized by neurological, psychiatric and endocrinological features, which include hypotonia, psychomotor retardation, feeding difficulties during infancy and excessive eating after the age of 16-24 months with morbid obesity, compulsive behavior, temper tantrum, short stature, hypogonadism [47]. The cause of PWS is represented by the absence of expression of paternal genes from chromosome 15q11.2–q13 through different mechanisms: a deletion of chromosome 15q11.2–q13 of paternal origin (65–75%), a maternal uniparental disomy (20–30%), an imprinting defect of 15q chromosome (1–3%) [47].

The prevalence of epileptic seizures in PWS varies in different studies between 0 to 35%, mainly febrile seizures, focal and generalized tonic-clonic seizures [48]. Other types of seizures are rarely reported, such as complex partial seizures, atypical absence, staring spells, and myoclonic, tonic, hemiclonic and atonic seizures, [48, 49, 50, 51, 52]. Focal epileptiform activity and EEG seizures are seen in individuals with no history of epilepsy, especially in the young age group [48].

All types of AED have been used, including CBZ, LTG, TPM, PHB, LVT, PHT, CLB [48]. Valproic acid, as well pregabalin, gabapentin, carbamazepine and corticosteroids, should be used with caution in these patients due to its high risk of weight gain. The progosis of epilepsy in PWS is favorable, a good control of seizures being achieved with monotherapy [53, 54].

## 5 22q11.2 deletion syndrome

22q11.2 deletion syndrome (22q11.2DS) is the most frequent interstitial deletion syndrome, with a prevalence of 1:4000 live newborns [55]. The main features include congenital heart malformations, palatal defects, hypoparathyroidism with hypocalcemia, dysmorphic facial features, intellectual disability, neuropsychiatric diseases (schizophrenia, autism spectrum disorders etc.), thymic hypoplasia/involution and T-cell anomalies [55, 56]. Although epilepsy was considered as a rare manifestation of 22q11.2DS, more recent studies reported a prevalence of epilepsy in this syndrome ranging from 3.4% to 15,2% [57, 58], and, also, an association of this syndrome with generalized epilepsy or juvenile myoclonic epilepsy [58]. Additionally, different brain malformations were reported as relatively common in 22q11.2DS, and represent an important cause of epilepsy [58], refractory at all types of AEDs. For generalized epilepsies, LVT or VPA can be used with good effect. Another risk factor for seizures in patients with 22q11.2DS is represented by hypocalcemia. Calcium plays an important role in neuronal excitability, but also in neuronal development and function [59]. Recent studies showed that neonatal seizures secondary to hypocalcemia in children with 22q11.2DS were associated with a subsequently moderate or severe intellectual disability [59]. Thus, a rapid correction of hypocalcemia in these children is mandatory in order to prevent both seizures and seizure related cognitive delay.

In adults with 22q11.2DS an important risk factor for epileptic seizures is psychiatric medication. The antipsychotic drugs most commonly associated with seizures were clozapine, phenothiazines, risperidone, and haloperidol [60]. Among antidepressants, clomipramine was reported with a higher risk of seizures [60].

## 6 Ring chromosome 20

Ring chromosome 20 is a rare genetic syndrome associating epilepsy, intellectual disability and behavior problems. The prevalence of the syndrome is around 1 in 30 000‐60 000 births, with mainly sporadic cases [61, 62]. Taking in account the origin and structure of ring 20, there are two patient groups described: one group with mosaic ring 20 and no detectable deletions and a non-mosaic group with a deletion at one or both ends of the chromosome 20 [63]. A difference has been observed between the two patient groups, regarding the epileptic phenotype, the age of seizure onset was significantly lower in non-mosaic group [63].

The most common epileptic seizures type are partial complex seizures, and, in some cases non-convulsive status epilepticus has been reported. Childhood onset seizures consists of focal motor or dyscognitive seizures. Adolescence onset is usually associated with a milder evolution and no cognitive delay [64, 65].

The mechanism underlying the epilepsy development in ring 20 syndrome is still unknown; the proposed theories include: haploinsufficiency of candidates genes (i.e. *CHRNA4* and *KCNQ2*), gene expression silencing by a telomere position effect, the deleterious effect of ring instability [63, 66, 67].

In the great majority of cases the seizures are refractory to all antiepileptic drugs, both as monotherapy or in different combinations, with a deleterious effect on cognitive development of these children. The epilepsy outcome seems to be proportional to the percentage of ring chromosomes seen in the mosaic karyotype analysis, and the age of seizure onset [65]. Some authors recommend, as the first therapeutic choice, administration of VPA and LTG, in combination [64]. It is noteworthy that in some patients, the use of TPM or CBZ combined with LVT, led to a worsening of the seizures [65].

## 7 Wolf-Hirschhorn syndrome

Wolf-Hirschhorn syndrome (WHS) or chromosome 4p deletion is a rare genetic condition characterised by global developmental delay, dysmorphic features, and various malformations.

WHS frequency is approximately 1:50,000 births to 1:95,000 births [68, 69]. This syndrome is caused by partial deletions of chromosome 4 short arm, 4p16.3 being a critical region [70]. The genetic defects in most patients are *de novo* deletions; however in some patients the 4p deletions are generated by unbalanced translocations or other chromosomal rearrangements [71].

Epileptic seizures are present in over 90% of patients with WHS, generalised tonic-clonic and complex partial seizures beeing the most common reported seizures; some patients develop atypical absences by the age 1 to 6 year-old [72, 73]. Approximately 40-50% of cases with WHS present also status epilepticus [72, 73].

As in other chromosomal deletion syndromes, the pathogenetic mechanisms of epilepsy are largely unknown. Two critical regions for this syndrome were detected, WHSCR1 and WHSCR2 [74,75]. Candidate genes for epilepsy phenotype were identified in these regions, such as Wolf-Hirschhorn Syndrome Candidate 1 gene (*WHSC1*, current name Nuclear Receptor Binding SET Domain Protein 2 - *NDS2*) and Leucine Zipper And EF-Hand Containing Transmembrane Protein 1 gene (*LETM1*), respectively [75, 76, 77]. *WHSC1* codes for a histone methyltransferase with H3K27me methyltransferase activity, expressed in early development and considered to function as a transcription regulator that binds DNA. LETM1, is involved in calcium signaling and homeostasis by encoding a member of the EF‐hand family of calcium‐ binding proteins [77].

Different antiepileptic drugs, as monotherapy or in different combination, have been proposed. In a study on 300 patients with WHS, the most efficacious drugs were LVT and CLB, followed by phenobarbital and VPA; on the other hand, CBZ, OCZ and PHT had the worst effect on seizures control [73]. Regarding the side effects, evaluated after the frequency of drug discontinuation, LVT, CLB and diazepam were well tolerated, whereas CBZ, OCZ, TPM and phenobarbital had a poor tolerability.

## 8 Fragile X syndrome

Fragile X syndrome (FXS) is the most common cause of intellectual disability, affecting 1 in 2500 – 4000 males. In most patients, FXS is caused by triplet (CGG) repeat expansion mutation of the 5′-untranslated region of the fragile X mental retardation 1 (*FMR1)* gene localized in Xq27.3. FXS full mutation alleles contain 200 or more copies of the triplet repeat that are hypermethylated and thus inactivates FMR1 gene by transcriptional silencing [78, 79]. In rare cases (less than 1% of the patients), point mutations and partial or complete FMR1 gene deletions lead to FXS phenotype [80].

FXS is characterised by developmental delay, macrocephaly, dysmorphic features, behavior problems (autism, hyperkinesia etc), macroorchidy. Epilepsy was reported in 10 to 40% of patients with FXS, both males and females [78].

FXS is characterized by altered neuronal excitability [81] that leads to hyperactivity hypersensitivity to sensory stimuli and epilepsy [82]. The mechanisms that lead to this increased neuronal excitability in the absence of FMRP are not elucidated. Recently, Gross et al (2011) demonstrated in a *Fmr1* KO mouse model, that FMRP is involved in regulation of translation and protein expression of the A-type potassium channel Kv4.2 [83]. Functional deletion of Kv4.2 has been previously reported in temporal lobe epilepsy in humans [84], thus Kv4.2 dysregulation might represent the link between FXS and epilepsy.

The most common type of seizures are partial seizures, especialy in association with centrotemporal spikes, resembling benign rolandic epilepsy [78]. Generalised tonic-clonic seizures were also noted in these patients,and, with a lower frequency, status epilepticus. The most used antiepileptic drugs are CBZ for partial seizures and VPA for generalised seizures or for cases who do not responded to CBZ [78]. In patients with no seizure control with these two drugs, LTG can represent an option.

## 9 Monosomy 1p36

Chromosome 1p36 deletion syndrome is a severe neurodevelopmental disorder characterized by intellectual disability and multiple congenital anomalies [85]. The prevalence of 1p36 monosomy is 1:5000 newborns [86, 87], making this condition the most common terminal deletion syndrome. Chromosome 1p36 monosomy can be generated by terminal or interstitial deletions, derivative chromosomes or complex chromosomal rearrangements. 1p36 deletions show an important size variability and no common breakpoints.

The typical clinical findings of this syndrome include moderate to severe global developmental delay, characteristic craniofacial abnormalities, and hypotonia. Additional common features are motor skills and language impairment, epilepsy, congenital heart defects, hearing loss and ocular problems [85, 88, 89].

Epilepsy is a clinical feature occuring in approximately 50–60% of cases [87, 89]. The seizures types described in 1p36 monosomy are highly variable and include infantile spasms, partial or generalized tonic–clonic, myoclonic, typical and atypical absence seizures and atonic seizures [85, 89, 90]. Similarly, the EEG findings are variable, focal and multifocal spikes, hypsarhythmia, and asymmetry of slow activity being reported [88, 91].

The pathogenesis of epilepsy in this syndrome is still unknown, due to the high gene density of 1p36 and to the variability of deletion sizes and genomic breakpoints. Diferrent critical region for epilepsy were delineated [92, 93, 94]. Haploinsufficiency of several genes was considered a potential contributor to seizures , such as *GABRD (*delta subunit of the gamma-amino butyric acid receptor gene)*, KCNAB2* (voltage-gated potassium channel subunit beta-2) *gene* and *SKI* (SKI proto-oncogene) [89,95].

Various AEDs are reported in the literature for epilepsy treatment in 1p36 monosomy syndrome: high doses of oral steroids, VPA, LEV, VBG, ESM, PHB, association of VPA with ACTH or multidrug combination. The prognosis of epilepsy is reported as usually favourable; however a percentage of patients experience drug resistant seizures [85, 89, 90, 95]. The onset of epilepsy with ISs, in 1p36 deletion syndrome, seems to be associated with a higher risk of developing refractory epilepsia [89, 91, 95].

## 10 Inv-dup(15) syndrome

Inverted duplication of chromosome 15 syndrome - inv dup(15) syndrome, isodicentric chromosome 15 syndrome, tetrasomy 15q syndrome - is caused by the presence of at least one supernumerary copy of 15q11.2-13.1, inherited from the mother, and which comprise PWS/AS critical region. The syndrome has an estimated incidence of 1 to 30,000 newborn babies [96].

The clinical presentation include early central hypotonia, moderate to profound developmental delay and ID, epilepsy, and autistic behavior [97]. There are two chromosomal mechanisms that leads to the characteristic clinical presentation of inv dup(15) syndrome: the presence of a maternal isodicentric 15q11.2-q13.1 supernumerary chromosome resulting in tetrasomy or hexasomy for 15q11.2-q13.1 (80% of cases) and a maternal interstitial 15q11.2-q13.1 duplication or triplication (20% of cases).

Epilepsy is present in more than 50% of the patients with inv dup(15); a wide variety of seizure can occur, including infantile spasms and myoclonic, tonic-clonic, absence, and focal seizures. Frequently, there are difficult to treat epilepsy forms associated with some degree of deterioration in cognitive skills and behaviour [98]. Various EEG abnormalities have been described, such as slow/sharp waves, and/or biphasic spikes-polyspikes, spike/wave complexes, and an excess of fast activity mainly over the fronto-temporal areas [98, 99].

An efficient control of IS can be achived by administration of ACTH in high doses [100]. VPA, CBZ, LTG and rufinamide proved to be the most effective as sole drug or in associations. The antiepileptic terapy can be selected based on type of seizures at onset: in cases with atypical absence, VPA can be use with good effect, whereas in patients with tonic seizure, CBZ can be the first choice [99].

## 11 Conclusions

Epilepsy associated to different chromosomal developmental syndromes, raises specific problems of treatment and prognosis. Thus, it is very important to choose the proper AEDs for specific types of epilepsy. Such examples are the use of VGB and steroids or ACTH in ISs associated to DS and use of VPA and LVT in AS, DS with myoclonic seizures (adult patients) and in generalized epilepsies associated with 22q11.2DS. Some AEDs should be avoided because they can exacerbate the seizures, such as CBZ and OCZ in patients with AS or in those with myoclonic seizures. Also, we should take into consideration the other clinical features of these patients which can be aggravated by some AEDs: TPM and VPA can increase the cognitive deficit, and VPA should be avoided in syndromes associating obesity such as PWS and DS. For patients with refractory epilepsy, like in AS, ketogenic diet or low-glycemic index treatment can be useful.

In conclusion, as in all epileptic syndromes, the antiepileptic therapy in chromosomal developmental syndromes should be better standardized and personalized in order to obtain a good control of seizures and to avoid or minimize the side effects, especially those with neuropsychiatric impact.

These chromosomale syndromes should be taken into consideration in evaluation of a child with first episode of epileptic seizure, especially in association with some specific features such as intellectual disability, dysmorphic features, or behavioral problems.
